# Chronic Maxillary Rhinosinusitis of Dental Origin: A Systematic Review of 674 Patient Cases

**DOI:** 10.1155/2014/465173

**Published:** 2014-04-08

**Authors:** Jerome R. Lechien, Olivier Filleul, Pedro Costa de Araujo, Julien W. Hsieh, Gilbert Chantrain, Sven Saussez

**Affiliations:** ^1^Laboratory of Anatomy and Cell Biology, Faculty of Medicine, UMONS Research Institute for Health Sciences and Technology, University of Mons (UMons), Avenue du Champ de Mars 6, B7000 Mons, Belgium; ^2^Laboratory of Phonetics, Faculty of Psychology, Research Institute for Language Sciences and Technology, University of Mons (UMons), B7000 Mons, Belgium; ^3^Laboratory of Neurogenetics and Behavior, Rockefeller University, 1230 York Avenue, New York City, NY 10065, USA; ^4^Department of Otorhinolaryngology, Head, and Neck Surgery, CHU Saint-Pierre, Faculty of Medicine, Université Libre de Bruxelles (ULB), B1000 Brussels, Belgium

## Abstract

*Objectives*. The aim of this systematic review is to study the causes of odontogenic chronic maxillary rhinosinusitis (CMRS), the average age of the patients, the distribution by sex, and the teeth involved. *Materials and Methods*. We performed an EMBASE-, Cochrane-, and PubMed-based review of all of the described cases of odontogenic CMRS from January 1980 to January 2013. Issues of clinical relevance, such as the primary aetiology and the teeth involved, were evaluated for each case. *Results*. From the 190 identified publications, 23 were selected for a total of 674 patients following inclusion criteria. According to these data, the main cause of odontogenic CMRS is iatrogenic, accounting for 65.7% of the cases. Apical periodontal pathologies (apical granulomas, odontogenic cysts, and apical periodontitis) follow them and account for 25.1% of the cases. The most commonly involved teeth are the first and second molars. *Conclusion*. Odontogenic CMRS is a common disease that must be suspected whenever a patient undergoing dental treatment presents unilateral maxillary chronic rhinosinusitis.

## 1. Introduction

Chronic rhinosinusitis (CRS) is the most frequent pathology in USA, since it affects 33.7 million people each year [1], representing nearly 14% of the American population [2]. According to various reports, a dental origin is found in 5 to 40% of cases of chronic maxillary rhinosinusitis (CMRS) [1, 3, 4]. CMRS is defined by the presence of ongoing rhinosinusal symptoms for at least 12 weeks [5, 6]. Its incidence is consistently growing and it is more frequent among women [7]. The majority of CMRS patients are between 30 and 50 years old. From an anatomic perspective, maxillary sinus are air-filled cavities situated laterally to the nasal fossae and communicate with them through an ostium which is approximately 4 millimetres in diameter and vulnerable to occlusion during mucosal inflammation [8]. The maxillary sinus anatomical relationships involve the dental roots inferiorly, explaining the easy extension of the infectious processes from some teeth to the maxillary sinus [3, 9]. The paranasal sinuses and the whole nasal fossae are covered with a ciliated pseudostratified epithelium. The essential role of this epithelium is the secretion of respiratory mucus and its movement to the nasopharynx, ensuring elimination of sinus secretions towards the nasal fossa. Normal mucociliary clearance requires an adequate permeability of the sinus ostium as well as good secretory and ciliary functions [10]. From a pathophysiological point of view, CMRS is due to a temporary and reversible mucociliary dyskinesia [11], which could be favoured by several factors: gastroesophageal reflux disease [12], atmospheric pollution [13], smoking [14], nasosinusal polyposis [15], arterial hypertension [15], dental infections, anatomic malformations such as septal deviations, concha bullosa, allergic reactions, and immune deficits [16–20]. Odontogenic CMRS occurs when the Schneiderian membrane is irritated or perforated, as a result of a dental infection, maxillary trauma, foreign body into the sinus, maxillary bone pathology, the placing of dental implants in the maxillary bone, supernumerary teeth, periapical granuloma, inflammatory keratocyst, or dental surgery like dental extractions or orthognathic osteotomies [3, 21]. Among the CMRS induced by foreign bodies, one might distinguish between exogenous or, less frequently, endogenous foreign bodies. The most frequent types of exogenous foreign bodies are endodontic material used in dental obturation [9]; these foreign bodies can trigger an inflammatory response and an alteration of the ciliary function [22, 23]. A CMRS caused by a dental infection can take two different routes to spread the infection. It can extend into the sinus through the pulp chamber of the tooth, causing an apical periodontitis. If the “tooth height” is altered due to a chronic infection and destruction of the tooth socket, we call it a marginal periodontitis.

Once the drainage is compromised by mucosal oedema, sinus infection may start involving various microorganisms. In bacteriological studies, it is well recognised that anaerobes can be isolated in up to two-thirds of patients who have CRS, mostly in the setting of a polymicrobial infection [24]. *α*-hemolytic* Streptococcus *spp., microaerophilic* Streptococcus *spp., and* Staphylococcus aureus* are predominant aerobes and the predominant anaerobes are* Peptostreptococcus *spp. and* Fusobacterium *spp. [3]. There is a difference between the bacteriology of odontogenic CMRS and that of other cases; however, in clinical practice, taking an uncontaminated bacteriological sample might turn out to be difficult. In addition, fungal superinfections are frequent and increased by immunodeficiency, diabetes mellitus, sinus radiotherapy and, excessive antibiotic and corticosteroid use [10, 17, 25]. Dental amalgams may sometimes contain minerals such as zinc oxide, sulphur, lead, titanium, barium, calcium salts, and bismuth that may accelerate fungal growth [17]. Microbiological findings often reveal* Aspergillus fumigatus* and, more rarely,* Aspergillus flavus*, which may be much more aggressive [17, 25, 26]. Different theories are put forward to explain those* aspergillus* superinfections. Following a French etiologic hypothesis, an* Aspergillus* infection would also be odontogenic, requiring an oroantral fistula to allow sinus contamination. Other hypotheses favour a mixed origin or strict aerogenic contamination via heavy spore inhalation over an extended period of time [22, 27]. CMRS is clinically characterised by a variable association of symptoms including anterior or posterior, unilateral or sometimes bilateral discharge (purulent, watery, or mucoid), sinus or dental pain, nasal obstruction, hypo- or anosmia facial headaches that intensify in the evening while bending, halitosis, and occasionally coughing [17]. Even if there is no significant difference between classic and odontogenic CMR, anterior discharge, sinus pain, nagging pain of the upper teeth of the damaged side that increases during occlusion and tooth mobilisation, and halitosis seem to be more frequent in the latter [21, 25]. Percussion of the causal tooth may reveal an abnormal sensitivity, unless endodontic filling has been performed. Most cases are unilateral, although bilateral cases have been described as well [7]. The time interval between symptoms onset and the causal dental procedure may be highly variable: according to Mehra and Murad, 41% of patients developed CMRS in the following month, 18% between one and three months after the procedure, 30% from three months to one year, and 11% of patients after more than one year [8]. Computed tomography (CT) of the sinus is essential. Some authors also recommend the Valsalva test for diagnosing an oroantral communication [10]. Most of the literature concerning odontogenic CMRS consists of either prospective or retrospective reports, and the guidelines on how to deal with the disease are often based on expert opinions.

## 2. Materials and Methods

### 2.1. Aim

The aim of this review is to define the aetiologies of odontogenic CMRS and the teeth involved.

### 2.2. Literature Search and Data Extraction

**Figure 1:**
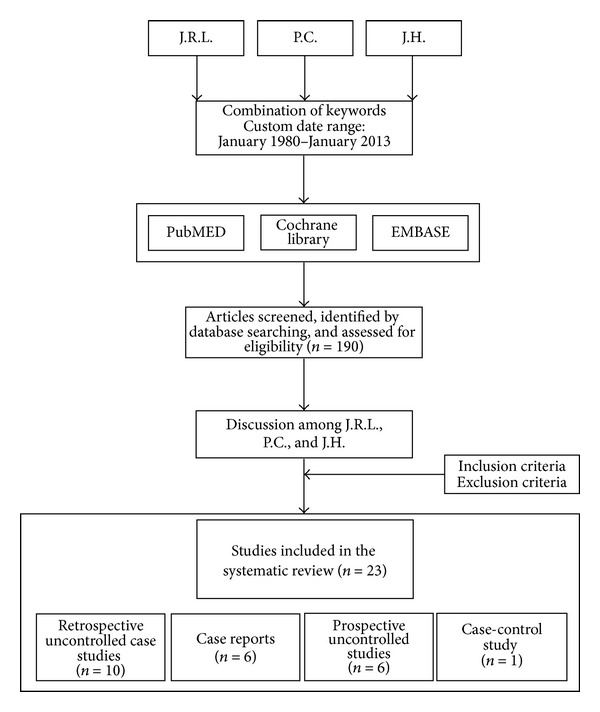
Flow chart shows the process of article selection for this study.

The literature was reviewed independently by three different authors (Jerome R. Lechien, Pedro Costa de Araujo, and Julien W. Hsieh) to minimise inclusion biases. The authors were not blinded to the study author(s), their institutions, the journal, or the results of the studies. The search for articles was done through PubMED, Cochrane Library, and EMBASE (Figure 1). It included all articles written in English, French, and other languages and published between January 1980 and January 2013. We focused only on published papers. The keywords used were “odontogenic, chronic, maxillary sinusitis, dental, cyst, foreign body, iatrogenic, and periodontitis.” The initial 190 references (including case reports, retrospective and prospective studies) were manually sorted to extract all descriptions of patients meeting the diagnostic criteria of chronic maxillary rhinosinusitis proposed by the European position paper on rhinosinusitis and nasal polyps 2012 [6]. Methodologic quality was assessed by the authors to determine the validity of each study. When important data were missing in some studies, the first author (Jerome R. Lechien) tried to contact the authors to obtain the additional information. In addition, references were obtained from citations within the retrieved articles. To avoid multiple inclusions of patients, we checked for the age, gender, author, and geographic area, whenever they were available. If a patient was described in more than one publication, we used only the data reported in the larger and more recent publication. Patient demographic data, age, gender, and the teeth involved in odontogenic cases were only recorded on the basis of individual data; if it was impossible to obtain these data from the authors, they were considered missing.

### 2.3. Inclusion and Exclusion Criteria

The diagnosis of CMRS was based on;

1. the presence of ongoing rhinosinusal symptoms for at least 12 weeks secondary to a clearly identified dental cause (including traumatic, iatrogenic, tumour, and dental infectious);
2. the diagnosis of CMRS should be confirmed by computed tomography or by panoramic radiography.

Concerning periodontal infections, they were defined as clearly identified infections around the teeth that were concomitant of CMRS. Immunocompromised patients, cases of acute and subacute rhinosinusitis, and unclear causes of dental origin and cases where the type of rhinosinusitis is not clear were excluded.

## 3. Results

**Figure 2:**
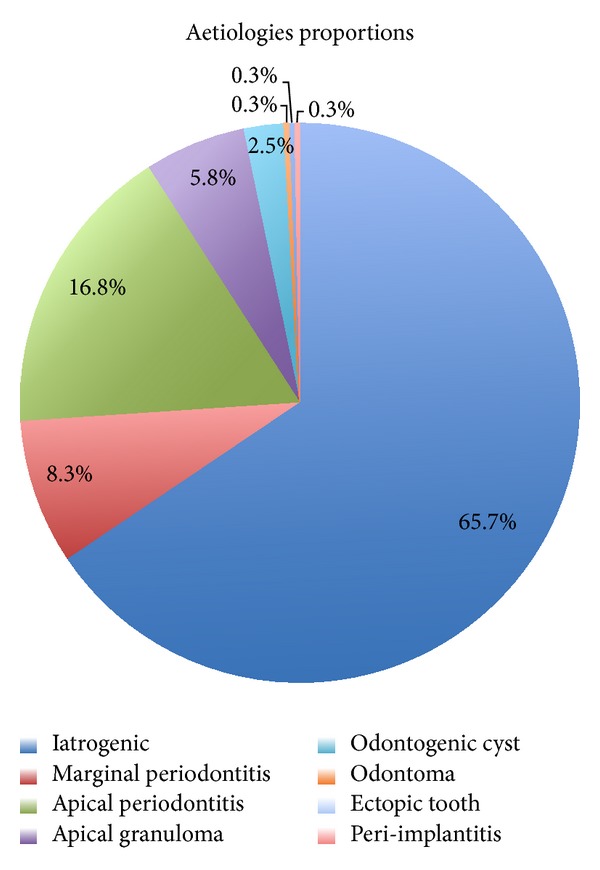
*Aetiology of odontogenic CMRS.* The main cause of odontogenic CMRS is iatrogenic and accounts for 65.7% of cases. Apical periodontal pathologies (including apical periodontitis, apical granulomas, and odontogenic cysts) and marginal periodontitis follow them and account for 25.1% and 8.3%, respectively. Peri-implantitis, ectopic tooth, and odontoma remain rare causes of odontogenic CMRS.

**Figure 3:**
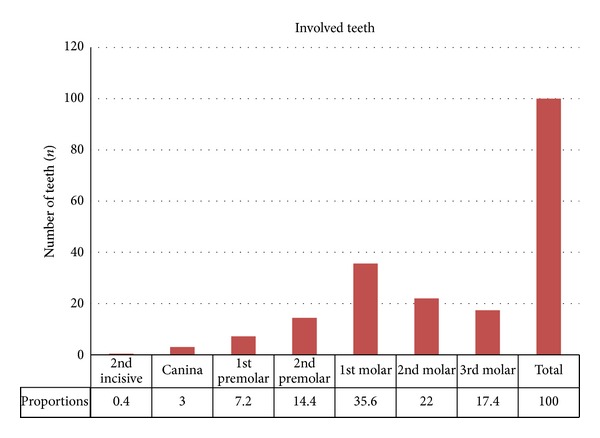
*Involved teeth.* The first and second molars were the most commonly affected teeth representing 35.6% and 22% of cases, respectively. They are followed by the third molar (17.4%), the second premolar (14.4%), and the first premolar (7.2%). The canina (3%) and the second incisiva (0.4%) remain rare and occasional.

Our database search yielded 190 articles. From these, we selected 23 articles, including 6 isolated case reports, 10 retrospective uncontrolled case studies describing 389 patients, 6 prospective uncontrolled studies describing 192 patients, and one case-control study describing 91 patients [11, 15, 22, 23, 26–44]. The description of all articles and ventilation of cases is displayed in Table 1. Among the 23 papers, 18 were published in English, two in both English and Spanish, and three in French. Fifty-four percent of all patients were women, and average patient age at diagnosis was 45.6 years (ranging between 12 and 81 years). The different aetiologies found in the literature search are summarized in Figure 2. Based on the 674 patients for whom it was displayed, iatrogenic causes were the most frequent, accounting for 65.7% of cases of described odontogenic maxillary rhinosinusitis. They included impacted tooth after dental care, artificial implants, dental amalgams in the sinus,and oroantral fistula. They were followed by apical periodontal pathologies, accounting for 25.1% of the cases. Apical periodontal pathologies include apical periodontitis (16.8%), apical granulomas (5.8%), and odontogenic cysts (2.5%). Unfortunately, the paucity of clinical descriptions limited the data of the involved teeth to only 236 cases. Nevertheless, as shown in Figure 3, the first and second molars were the most commonly affected teeth when reported, representing 35.6% and 22% of cases, respectively. They were followed by the third molar (17.4%) and the second premolar (14.4%).

## 4. Discussion

The aim of our study was to describe the aetiologies of odontogenic CMRS, the teeth involved, and age and sex distribution. To our knowledge, this paper is the first review studying the causes of CMRS. Further descriptions of CMRS causes were displayed in consecutive case series. In a case series of 70 patients with odontogenic CMRS, published by Lopatin et al., an exogenous foreign body from the teeth was found in 10 cases (14%), of which 7 dental amalgam fillings and 3 dental packings, and an endogenous foreign body (i.e. a tooth root) in 11 cases [30]. Thirty-nine patients (56%) also presented an oroantral fistula. Although rare, the foreign body was sometimes inserted in the sinus through trauma or accident [32]. In another case series of 125 patients suffering from odontogenic CMRS, the main aetiology was periapical chronic periodontitis (79% of patients), followed by complications of endodontic treatment (21% of cases) [32]. In addition, in two prospective studies of Melen et al. and Lindahl et al., most cases of CMRS were secondary to a dental infectious process such as marginal periodontitis and apical diseases [22, 39]. We compared the results of their studies with ours, specifically looking at aetiologies. Our results are consistent with the study of Lopatin et al., showing a majority of iatrogenic causes in comparison with infectious aetiologies. Our results in favor of the iatrogenic cause can be explained in part by the high proportion of studies reporting only a large number of iatrogenic etiology [15, 27, 29, 34, 37]. However, patient selection criteria were not described in most of the studies. Therefore, we were unable to control for selection bias, and our study may be subject to under- and overreporting bias. Finally, in a case series written by Krause et al., focusing on the foreign bodies found in any of the sinus, 60% of all foreign bodies were found to be iatrogenic and 25% of industrial accidents [45]. The sinuses affected were mainly the maxillary (75%) and frontal sinus (18%), foreign bodies in ethmoidal or sphenoid sinus being rare. Several studies found in the literature are limited by different biases. So the size of the clinical series is often relatively small, which may allow for undetected infrequent variants, and the retrospective design of the studies included did not let us make incidence estimations. Putting these limitations aside, the larger size of the sample studied allows for a better description of the pathology than what could be made based on a single case series, something crucial for a frequently overlooked condition. Concerning the gender distribution, our data show that women (57%) were slightly more affected by odontogenic CMRS than men (43%). Most clinical series are also characterized by a ratio in favour of women [29, 32, 33]. Among the dental characteristics found in the literature, information about the teeth involved is rare. Indeed, apart from the study of Lopatin et al. who reported involvement of the third molar, only three publications accurately investigated the teeth involved. The prospective study from Melen et al. shows that the most commonly involved teeth are the first (40.6%) and second molars (24.6%) [22]. Even with a smaller sample, Andric et al. observed similar proportions in their retrospective analysis where first and second molars account for 42% and 35%, respectively [37]. Finally, Lindahl et al. reported a higher proportion of the first molar (38%), followed by the second premolar (24%) and second molar (22%) [39]. These results can easily be explained by the preferential anatomical relationships between the floor of the maxillary sinus and the various teeth concerned (premolars, first and second molars). These proportions are similar in our study. However, our work is limited by the retrospective nature of most reports, which may include selection bias in the overall description.

The diagnosis of unilateral chronic maxillary RS required systematic dental examination and sinus computed tomography (CT) [22]. CT, with reconstructions following the axial and coronal planes, classically reveals sinus filling or a chronic mucous swelling associated with a reaction to foreign body [46, 47]. Interestingly, secondary aspergillosis, which is often associated with dental foreign body and appeared as a luminal opacity, can be misinterpreted as calcified dental amalgam. Other types of sinus opacities include ectopic tooth fragments, calcified retention cysts, osteoma, condensing osteitis, calcified polyps, odontomas, osteosarcomas, cementomas, bone fibrous dysplasia, and metastases of carcinoma [10].

Odontogenic CMRS is managed by both a medical and surgical approach. The first step consists of addressing the dental pathology and the second is a functional endoscopic sinus surgery. Starting with the dental intervention allows for the elimination of the origin of the infection as well as the removal of any newly introduced foreign sinus material in the same sinus endoscopy. Usually, the stomatologist or dental practitioner repeats the endodontic treatment or proceeds to an extraction. Addressing the sinusal component with functional endoscopic sinus surgery allows for the removal of the foreign bodies with a curved aspiration or a curved forceps and opening the sinus cavities for a better drainage. Minimal invasive endoscopic sinus surgery [23] is safer [48], quicker [3], has less impact on the sinus mucus clearance, provokes less bleeding, and allows for a shorter hospitalisation time [49]. The endoscopic approach is also recommended to treat* Aspergillus* infections with the exception of invasive mycotic complications. Medical treatment is based on decongestants and antibiotics selected with bacterial cultures.

## 5. Conclusion

Odontogenic CMRS is a frequent ENT pathology. Our review summarized the current clinical knowledge about aetiologies, teeth involved, gender, and age of this clinical entity. This condition affects women slightly more than it affects men. Patients are relatively young, given the average age of 45 years. Iatrogenic cause is the most common aetiology, and thus medical and dental practitioners should keep it in mind whenever a patient presents unilateral RS after dental treatment. The first and second molars are the most affected teeth, and the diagnosis is based on a combination of nasal endoscopy and CT, which usually displays sinus filling and intraluminal opacity. Managing odontogenic CMRS requires collaboration between the ENT specialist, the dental practitioner, the stomatologist, and the radiologist. The treatment always starts with the dental treatment, and then the removal of the foreign body is achieved by endoscopic route. Even if a complete cure is achieved in most cases, clinical follow-up remains critical for this pathology.

## Figures and Tables

**Table 1:** General characteristics of the studies. General table describing the studies characteristics (including category of evidence following the European position paper on rhinosinusitis and nasal polyps 2007 recommendations [6]) number of cases, aetiology, middle age, sex, and the involved teeth. CA: category of evidence, NA: not available.

Authors	Year	Review	Language	Study design	CA	*n* tot	Aetiology	*n*	Middle age (ranged)	Sex F	Sex M	Tooth	*n*
Lindahl et al. [39]	1981	Acta Otolaryngol	English	Prospective case series	III	29	Marginal periodontitis	13	52	NA	NA	Canina	2
							Apical periodontitis	14	42			1st premolar	5
							Iatrogenia	2	40			2nd premolar	11
												1st molar	17
												2nd molar	10

Melen et al. [22]	1986	Acta Otolaryngol	English	Prospective case series	III	99	Iatrogenia Marginal periodontitis Granuloma apical	17 43 39	48	NA	NA	2nd Incisiva Canina 1st premolar 2nd premolar 1st molar 2nd molar 3rd molar	1 4 11 23 56 34 9

Fligny et al. [27]	1991	Ann Oto-Laryng	French	Prospective case series	III	14	Iatrogenesis	14	42 (22–60)	NA	NA	NA	

Lin et al. [40]	1991	Ear Nose Throat J	English	Retrospective case series	III	16	Iatrogenesis	16	11–60	4	12	Canina 1st molar 2nd molar 3rd molar	1 5 3 2

Thevoz et al. [28]	2000	Schweiz Med Wochenschr	French	Retrospective case series	III	10	Iatrogenesis	10	48	NA	NA	NA	

Doud Galli et al. [29]	2001	Am J Rhinology	English	Retrospective case series	III	14	Iatrogenesis	14	(21–80)	10	4	NA	

Lopatin et al. [30]	2002	Laryngoscope	English	Retrospective case series	III	70	Iatrogenesis Odontogenic cyst	60 10	(16–62)	NA	NA	3rd molar	26

Cedin et al. [31]	2005	Braz J Otorhinolaryngol	English	Retrospective case series	III	4	Iatrogenesis	4	NA	NA	NA	NA	

Nimigean et al. [32]	2006	B-ENT	English	Retrospective case series	III	125	Apical periodontitis Iatrogenesis	99 26	46 (12–81)	69	56	NA	

Selmani and Ashammakhi [33]	2006	J craniofac surgery	English	Prospective case series	III	13	Iatrogenesis	13	45 (26–81)	8	5	NA	

Ugincius et al. [15]	2006	Stomatologija	English	Retrospective case series	III	136	Iatrogenesis	136	NA	NA	NA	NA	

Macan et al. [41]	2006	Dentomaxillo- facial Radiology	English	Case Report	III	1	Iatrogenesis	1	61	1	0	NA	

Srinivasa Prasad et al. [42]	2007	Indian J Dent Res	English	Case Report	III	1	Ectopic tooth	1	45	1	0	3rd molar	1

Mensi et al. [34]	2007	OOOOE	English	Case Control	IIB	91	Iatrogenesis	91	NA	NA	NA	NA	

Costa et al. [23]	2007	Oral and Maxillofacial Surgery	English	Prospective case series	III	17	Iatrogenesis Odontogenic cyst Peri-implantis	8 7 2	NA	NA	NA	NA	

Crespo del Hierro et al. [35]	2008	Acta Otorrinolaringol Esp	Spanish/English	Case Report	III	1	Odontoma	1	24	1	0	NA	

Rodrigues et al. [11]	2009	Med Oral Patol Oral Cir Bucal	English	Case Report	III	1	Iatrogenesis	1	62	0	1	NA	

Bodet Augustí et al. [36]	2009	Acta Otorrinolaringol Esp	Spanish/English	Retrospective case series	III	10	Iatrogenesis	10	NA	NA	NA	NA	

Andric et al. [37]	2010	OOOOE	English	Retrospective case series	III	14	Iatrogenesis	14	40	5	9	1st premolar 1st molar 2nd molar 3rd molar	1 6 5 2

Hajiioannou et al. [26]	2010	J Laryngol Otol	English	Prospective case series	III	4	Iatrogenesis	4	NA	NA	NA	NA	

Lechien et al. [38]	2011	Revue medicale de Bruxelles	French	Retrospective case series	III	2	Iatrogenesis	2	36	2	0	NA	

Mohan et al. [43]	2011	National Journal of Maxillofacial Surgery	English	Case report	III	1	Ectopic tooth	1	28	1	0	3rd molar	1

Khonsari et al. [44]	2011	Rev St Chir Maxillofac	English	Case report	III	1	Osteoma	1	52	1	0	NA	

Note: the incidence of oroantral communication is estimated at 0,58% of all premolar and molar extraction (ref); one can assume that the incidence of oroantral fistula is even smaller, keeping in mind that some oroantral communications were treated immediately after their creation or healed spontaneously. ref = Punwutikorn J, Waikakul A.
